# Supplementary material for: The p53 and Calcium Regulated Actin Rearrangement in Model Cells
Source: Int J Mol Sci. 2022 Aug 13;23(16):9078. doi: 10.3390/ijms23169078 (PMC9408879; doi:10.3390/ijms23169078)
Supplement: Supplementary file 1 [file ijms-23-09078-s001.zip › ijms-1825320-supplementary.pdf]

Supplements

The p53 and Calcium Regulated Actin Rearrangement in Model Cells

Alexandra Hencz[1,2], Edina Szabó-Meleg[1], Muhammad Yaqoob Dayo[1], Ardora Bilibani[1], Szilvia Barkó[1], Miklós Nyitrai[1] and Dávid Szatmári[1,*]

[1]University of Pécs, Medical School, Department of Biophysics, Pécs, Hungary, H-7624

[2]University of Pécs, Medical School, Institute of Physiology, Pécs, Hungary, H-7624

[c] equal contribution

[*]To whom correspondence: Dávid Szatmári (david.szatmari@aok.pte.hu)

Key words: Ca^{2+}, actin, p53, GSN, JMY,

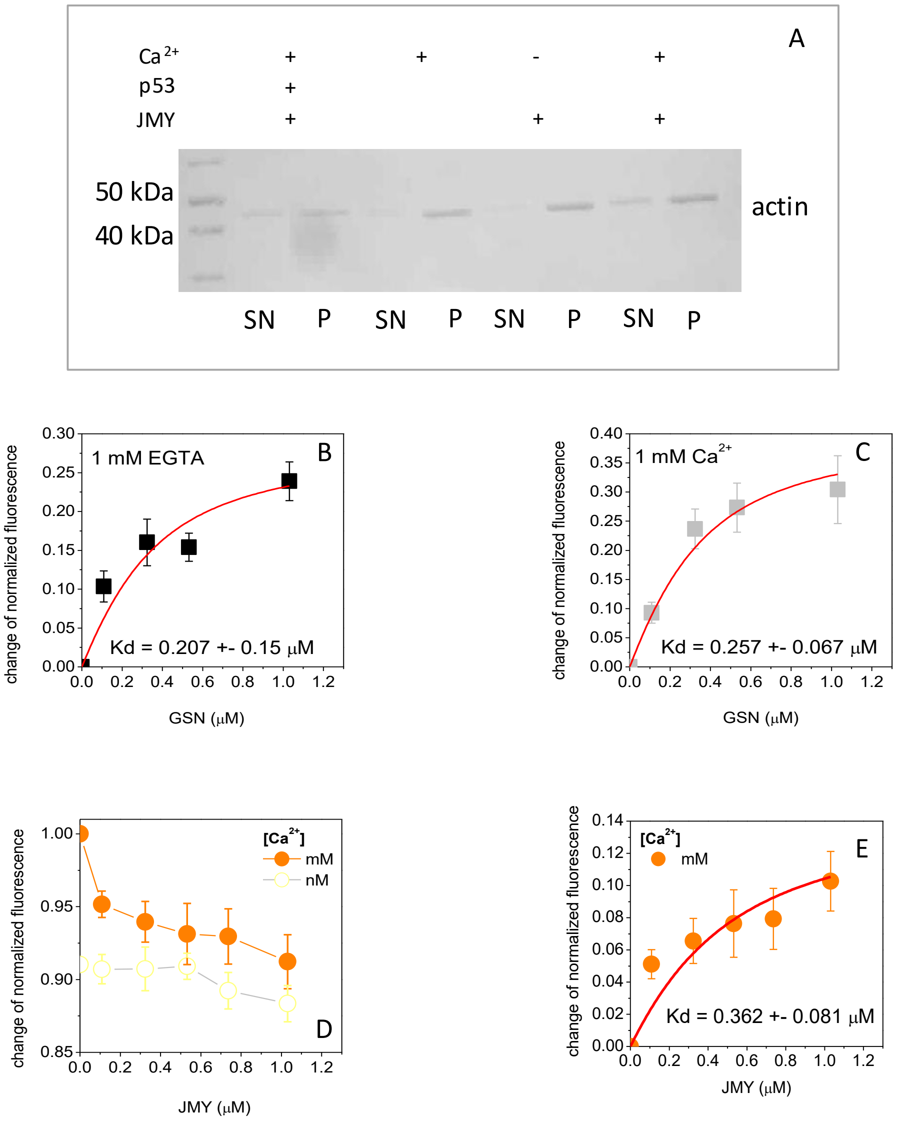

Figure S1. (**A**) SDS-PAGE of filamental actin (2 μM) cosedimentation with p53 (0.1 μM) and/or JMY (0.1 μM). (**B**) Normalized fluorescence change of 0.5 μM p53Alexa488p53 shows high affinity to the GSN (Kd= 0.207 ± 0.15 μM) which is almost the same as in case of (**C**) p53 dimers in the presence of Ca^{2+} (Kd= 0.257 ± 0.067 μM). (**D,E**) The p53 can bind to JMY in the presence (Kd= 0.362 ± 0.081 μM) but cannot in the absence of Ca^{2+}. The data presented were derived from at least 3 independent experiments. Values are displayed as the mean ± standard deviation.
